# Retraction

**DOI:** 10.1002/cam4.6773

**Published:** 2023-12-15

## Abstract

Zhao, W, Geng, D, Li, S, Chen, Z, Sun, M. LncRNA HOTAIR influences cell growth, migration, invasion, and apoptosis via the miR‐20a‐5p/HMGA2 axis in breast cancer. Cancer Med. 2018;7:842‐855. https://doi.org/10.1002/cam4.1353. The above article, published online on 23 February 2018 in Wiley Online Library (wileyonlinelibrary.com), has been retracted by agreement between the journal Editor in Chief, Stephen Tait, and John Wiley and Sons Ltd. The retraction has been agreed following an investigation into concerns raised by a third party, which identified image manipulation (duplication and rotation of the images) in Figure 2F, 4F, and 6F. As a result, the data and conclusions of the article are considered unreliable. The authors were informed of the decision to retract but remained unresponsive.

Zhao, W, Geng, D, Li, S, Chen, Z, Sun, M. LncRNA HOTAIR influences cell growth, migration, invasion, and apoptosis via the miR‐20a‐5p/HMGA2 axis in breast cancer. Cancer Med. 2018;7:842‐855. https://doi.org/10.1002/cam4.1353. The above article, published online on 23 February 2018 in Wiley Online Library (wileyonlinelibrary.com), has been retracted by agreement between the journal Editor in Chief, Stephen Tait, and John Wiley and Sons Ltd. The retraction has been agreed following an investigation into concerns raised by a third party, which identified image manipulation (duplication and rotation of the images) in Figure 2F, 4F, and 6F. As a result, the data and conclusions of the article are considered unreliable. The authors were informed of the decision to retract but remained unresponsive.

